# Antimicrobial peptides targeting bacterial ribosome

**DOI:** 10.18632/oncotarget.5114

**Published:** 2015-08-07

**Authors:** Ivan B. Lomakin, Matthieu G. Gagnon, Thomas A. Steitz

**Affiliations:** Department of Molecular Biophysics and Biochemistry, Yale University, New Haven, Connecticut, USA

**Keywords:** Chromosome Section, antimicrobial peptides, ribosome, antibiotic resistance, protein synthesis

Antibiotics kill bacteria by inhibiting vital enzymes involved in cellular metabolism, including protein production. However, extensive and sometimes gratuitous usage of antibiotics has led to microbial resistance, which is constantly emerging against every antibiotic class, raising serious public health concerns and the urgency for the development of new antibacterial therapeutics.

Different strategies can be used to circumvent target-specific resistance, including finding new antibiotic targets and designing compounds with stronger affinity to known targets. Recently, we and our colleagues revealed the mechanism used by the proline-rich antimicrobial peptide (PrAMP) Oncocin (Onc112) to inhibit bacterial protein synthesis [1, 2]. This mechanism illuminates a strategy, which has been conserved throughout evolution and is used by plants and animals against the ability of microorganisms to develop resistance. It inactivates protein synthesis in a clever manner: its binding site overlaps with three functional sites of the bacterial ribosome, limiting dramatically the probability of appearance of resistance mutations.

Antimicrobial peptides (AMPs) are part of the host innate immune system to counter infection. AMPs not only permeabilize and damage microbial cellular membranes, but also can operate by entering the cells and inhibiting some of their vital intracellular targets. The latter is an attribute of a group of the PrAMPs synthesized by mammals and insects. It includes Oncocin, Drosocin, Pyrrhocoricin and Apidaecin from insects, and the cathelicidin-derived peptides Bactenecin and PR-39 from mammals [3].

**Figure 1:**
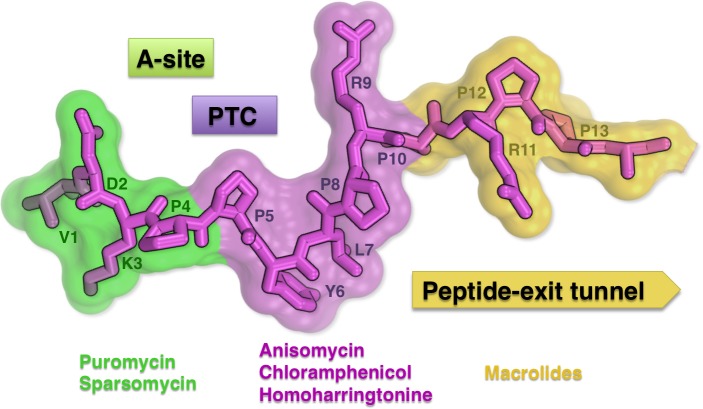
Structure of Onc112 bound to the bacterial ribosome Functional sites of the 50S subunit and antibiotics that bind to them are colored in green (A site), magenta (peptidyl transferase centre) and yellow (peptide-exit tunnel).

Recent data have shown that Apidaecin, Oncocin and Bactenecin inhibit protein synthesis and bind to the 70S ribosome approximately 50-fold stronger than to DnaK, which was previously identified as their main target [4, 5]. We characterized the primary target site for Onc112 by determining the crystal structure of the Onc112-70S ribosome complex [1]. Remarkably, unlike most known antibiotics, Onc112 interacts with not just one, but three functional sites of the ribosome simultaneously. This peptide forms a 34-Å-long plug that blocks access to the A and P sites of the 50S ribosomal subunit. Its N-terminus binds near the peptidyl transferase centre (PTC), where it interferes with both the A- and P-site tRNAs. The middle part of Onc112 occupies the A-site cleft in the PTC located at the peptide-exit tunnel entrance. The C-terminus of Onc112 binds inside the upper chamber of the peptide-exit tunnel and blocks it completely (Figure 1). It is striking that the majority of antibiotics that target the peptide-exit tunnel of the 50S subunit of the ribosome bind in these areas [6].

The CCA end and the aminoacyl moieties of the A- and P-site tRNAs would clash with the N-terminus of Onc112. Sequences of the N-termini of Oncocin, Pyrrhocoricin, Metain and Metalnikowin share the VDKP motif, while Bactenecin and PR-39 have a proline-arginine rich N-terminus. This may reflect different types of the peptide's N-terminal interactions with the 23S rRNA, which are required for binding and/or positioning of the peptide in the peptide-exit tunnel of the ribosome.

The middle part of Onc112 PYLPRP (Figure 1) binds the PTC—an extremely conserved region of the ribosome. Antibiotics bound to this region interact with the universally conserved nucleotide U2504 of the 23/25S rRNA, which discriminates between bacterial and eukaryotic binding-specificity [6, 7]. However, Phe6 of Onc112 is located too far from U2504, suggesting that Onc112 may bind the PTC pocket of eukaryotic ribosome as well. Motifs similar to PYLPRP are also present in other PrAMPs like Pyrrhocoricin, Metain, Metalnikowin, Bactenecin and PR-39. We propose that all these PrAMPs comprise a ribosome-binding group that inhibits bacterial and probably eukaryotic protein synthesis.

The C-terminal part of Onc112 binds to what is known as the macrolide binding site of the 50S subunit (Figure 1) [6]. Side chains of Arg9 and Arg11 extend for about 16 Å and block the upper chamber of the peptide-exit tunnel [1]. These and aforementioned interactions of Onc112 will prevent the binding of the tRNA to the A site of the 50S subunit, inhibit peptide bond formation and translocation of the nascent peptide through the tunnel, resulting in the accumulation of 70S ribosomes stalled on the initiation codon and inhibition of protein synthesis.

Our structural studies provide valuable information for understanding the mechanism of interaction between the peptide and its target. It shows that ability to bind simultaneously to several active sites of the ribosome is a valid strategy to eliminate appearance of resistant mutations. This builds a platform for structure-based design of the next generation therapeutics and will promote additional structural studies of other PrAMPs inhibiting protein synthesis.

The most intriguing question that is raised from our work is how Onc112 is able to penetrate into the peptide-exit tunnel. It remains to be determined how unstructured peptides of at least 18 amino acid residues long can be inserted in the peptide-exit tunnel. Finally, the binding site for Onc112 within the ribosome is extremely conserved. Thus we may expect that some PrAMPs will inhibit eukaryotic protein synthesis if delivered in the cytoplasm. Very low toxicity of PrAMPs for human cells and development of new drug-delivery vehicles, which target only specific groups of cells and can carry a peptide, make ribosome binding PrAMPs promising candidates for the development of new antitumor drugs.
